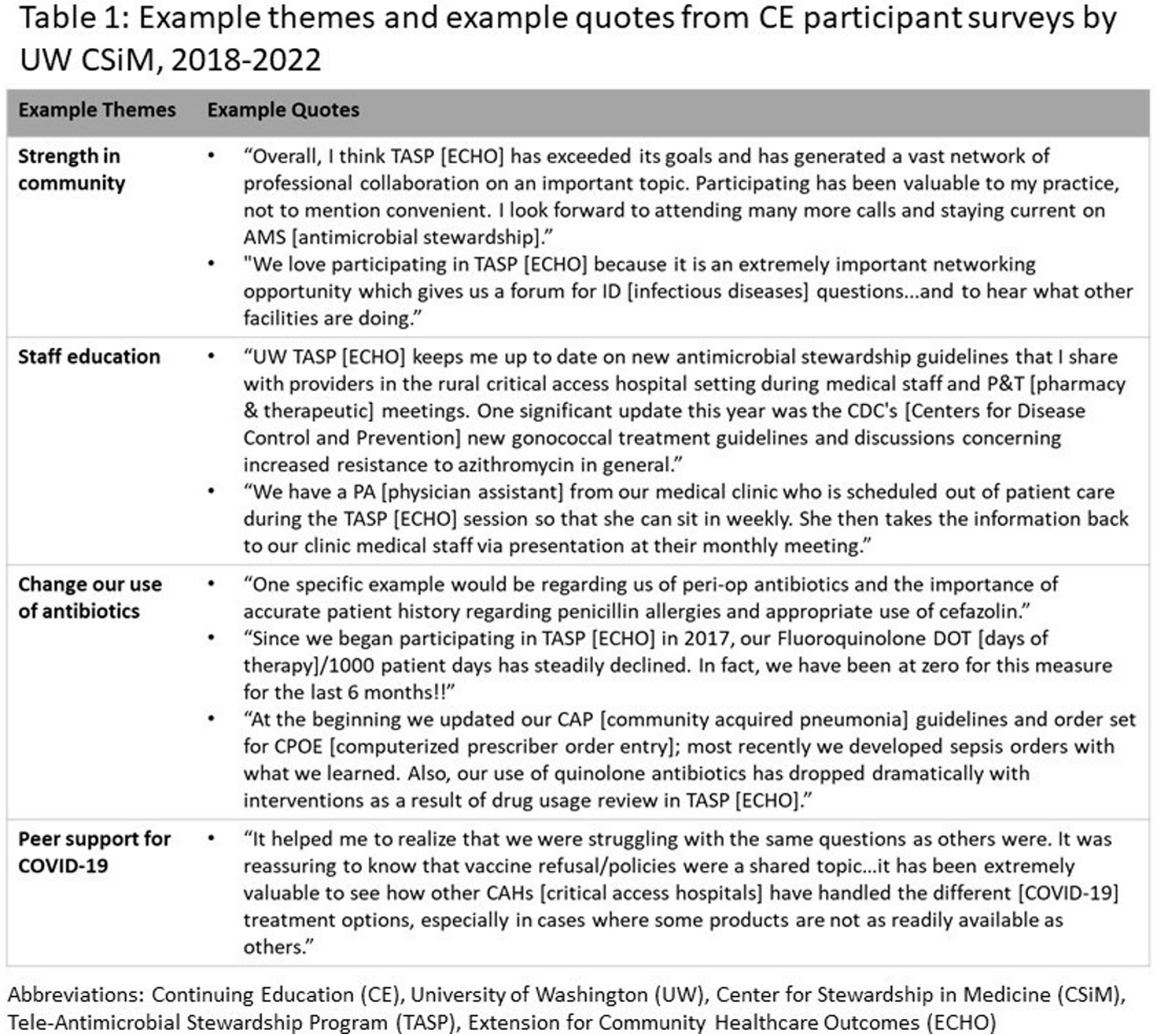# Qualitative Evaluation of an Antimicrobial Stewardship Tele-Mentoring Program in US Rural & Critical Access Hospitals

**DOI:** 10.1017/ash.2024.174

**Published:** 2024-09-16

**Authors:** Elizabeth Moore, Ellen MacLachlan, Natalia Martinez-Paz, Maria Bajenov, Rupali Jain, Jeannie Chan, John Lynch, Paul Pottinger, Zahra Kassamali Escobar, Chloe Bryson-Cahn

**Affiliations:** Applied Inference, LLC; University of Washington; University of Washington Medical Center; Harborview Medical Center; Fred Hutchinson Cancer Center

## Abstract

**Background:** The University of Washington (UW) Center for Stewardship in Medicine (CSiM) supports a tele-antimicrobial stewardship (AMS) program (TASP) using the ECHO approach (Extension for Community Healthcare Outcomes) in small, rural, and Critical Access Hospitals (primarily in the western U.S.) with education, mentoring, organizational capacity building, and a community of peers. To evaluate the continuing education (CE) component of UW-TASP ECHO, CSiM surveyed individuals receiving CE credits as part of the program. This survey was designed to track individuals’ satisfaction with the program and to assess the impact of UW-TASP ECHO on AMS in participating facilities. **Methods:** The CE participants’ survey was completed annually by individuals participating in UW TASP ECHO using online survey software. The survey included closed-ended and open-ended questions. Responses to open-ended questions were entered into Atlas.ti qualitative analysis software and coded iteratively according to themes that emerged. When a new code emerged partway through the coding process, earlier surveys were re-coded for the new code. Final codes were grouped into themes and sub-themes and quotes from each theme identified were summarized and attached to the theme and reported. **Results:** Data from three administrations of this survey were available: 2018-2019 (n=66); 2020-2021 (n=27); and 2021-2022 (n=30). These surveys were completed by a total of 95 individuals from 53 hospitals. Seven of these individuals completed a survey in each year, 14 completed a survey in two years, and 74 completed only one survey. Themes identified were COVID-19 support (including procedures and policies, being kept up-to-date, research summaries, and peer support), the antibiotic pocket guide developed by UW, strength in community, staff education, role of CSiM in developing/strengthening the AMS program at the facility, change in use of antibiotics, UW imprimatur, learning/growing as a healthcare provider, and importance for small, rural hospitals (see examples in Table 1). **Conclusions:** This qualitative analysis provides evidence from surveys of individuals participating in CE that UW TASP ECHO has had a meaningful impact in such domains as building a strong community among small, rural and critical access hospitals, educating staff, changing antibiotic use and providing peer support, among others.

**Figure:**